# A New Process for Efficient Recovery of Rhodium from Spent Carbonyl Rhodium Catalyst by Microreactor

**DOI:** 10.3390/ma16186271

**Published:** 2023-09-19

**Authors:** Lei Guo, Yifan Niu, Jianjun Hu, Shaohua Ju, Yongwan Gu, Wenjin Tan

**Affiliations:** 1 Faculty of Metallurgical and Energy Engineering, Kunming University of Science and Technology, Kunming 650093, China; gl_kmust@163.com (L.G.); 15234626355@163.com (Y.N.); 2 Key Laboratory of Unconventional Metallurgy, Ministry of Education, Kunming 650093, China; 3 National Local Joint Laboratory of Engineering Application of Microwave Energy and Equipment Technology, Kunming 650093, China; 4 Guiyan Group (Yimen) Co., Ltd., Yuxi 651100, China; 15750024102@163.com (J.H.); twj757@ipm.com.cn (W.T.); 5 State Key Laboratory of Complex Nonferrous Metal Resources Clean Utilization, Kunming 650093, China; 6 Kunming Institute of Precious Metals, Kunming 650106, China

**Keywords:** spent carbonyl rhodium catalyst, microreactor, rhodium, green recycling, response surface methodology

## Abstract

Triphenylphosphine acetylacetone carbonyl rhodium (ROPAC) is an important catalyst in the petrochemical industry, and its deactivated waste catalyst holds significant value for recovery. This study focuses on the existing forms of rhodium (Rh) in waste catalysts and the current status of traditional processes. A green, efficient, and continuous recovery technique was developed using a sealed stainless steel microchannel reactor. The influence of reaction temperature, reaction time, and phase ratio on the Rh recovery rate was investigated, and the process parameters were optimized using response surface methodology (RSM). The results indicate that the magnitude of the impact on the Rh recovery rate follows the order: reaction temperature > reaction time > phase ratio. The optimized process parameters were determined as follows: a reaction time of 29 min, a reaction temperature of 110 °C, and a phase ratio of 1:1, with a corresponding maximum recovery rate of Rh of 66.06%. Furthermore, secondary treatment was performed on the organic phase after primary recovery using the same process conditions, resulting in an overall Rh recovery rate of 95.6%, indicating satisfactory recovery efficiency. Moreover, the application of FTIR and ICP-OES analysis provided definitive evidence that the oxidative dissociation of the rhodium-phosphine chemical bond by H_2_O_2_ within ROPAC leads to the conversion of Rh^+^ into Rh^3+^. Subsequently, Rh forms chloroaquorhodium (III) complexes that enter the aqueous phase, enabling effective recovery of Rh.

## 1. Introduction

Butyl octanol is an important raw material for the synthesis of fine chemical products. Low-pressure carboxyl synthesis is the main industrial production process at present [1]. Its core catalyst is triphenylphosphine acetylacetone carbonyl rhodium (ROPAC) [2,3]. In 2018, the global production capacity of butanol and octanol was approximately 16.5 million tons/age, while China’s production capacity was 5.1 million tons/age, ranking first in the world [4]. As a core catalyst for production processes, ROPAC is irreplaceable. However, due to the inevitable presence of impurity chloride ions in synthesis gas, impurity chloride ions are toxic to the ROPAC catalyst [5]. They will coordinate and bind with rhodium, seriously affecting the speed of the olefin aldehyde reaction and ultimately leading to permanent deactivation of the catalyst, and the annual reported waste amount has reached over 1000 tons [6,7]. Due to the scarcity and high cost of rhodium (Rh) resources in the Earth’s crust, it is necessary to recover rhodium from ROPAC spent catalysts due to their high rhodium content [8].

Currently, the methods of recovering Rh from ROPAC waste catalyst in industrial production mainly include incineration [9], precipitation [10], extraction [11], adsorption [12], electrolysis [13], reduction, and oxidation [14,15]. Among the methods, the oxidation method is to use the oxidant to destroy the coordination of the ligand and the metal Rh in the catalyst under appropriate conditions, so that the Rh is separated from spent homogeneous catalysts in the form of Rh^3+^ or solid particles, and the following reaction occurs: 2RhH(CO)(PPh_3_)_3_ + 5O_2_ + 6H^+^→2Rh^3+^ + 2H_2_ + 2CO_2_ + 6Ph_3_PO + 2H_2_O [16]. In previous reports, Liu et al. (2003) pretreated the waste catalyst by roasting and reduction and then oxidized it with a mixed solution containing hydrogen peroxide (H_2_O_2_), hydrogen ions (H^+^), and chloride ions (Cl^−^) to obtain a water-soluble compound of Rh, and then further purified and recovered the precious metal [17]. Li et al. (2013) published a method for recovering Rh from waste catalysts containing Rh in hydroformylation reactions with H_2_O_2_. In the first step, the waste Rh liquid was treated with H_2_O_2_, and 90% of the Rh in the waste liquid was recovered [18]. Li et al. (2014) also used the H_2_O_2_ oxidation method to recover Rh from deactivated propylene hydroformylation catalyst and directly prepared RhCl(CO)(TPP)_2_ with Rh-containing waste catalyst solution as the starting solution, so that Rh was reconverted into Rh catalyst intermediate [19]. From the reported literature, it is evident that the combination of oxidation methods with other processes has achieved significant results in the recovery of Rh from spent catalysts. However, traditional Rh recovery processes are carried out in sealed, heated reactors, requiring the addition of a large amount of H_2_O_2_ for Rh recovery. This poses a great threat to worker safety due to the potential risk of gas overflow and prolonged reaction times. Furthermore, considering the high cost of Rh, it is crucial to ensure a high recovery rate during the recovery process of Rh from solutions containing spent catalysts in order to make the operation economically viable. Additionally, the discharge of unrecovered Rh into the environment poses considerable environmental hazards. Therefore, the development of a safe, efficient, environmentally friendly, and high-yield method for recycling waste ROPAC catalysts holds significant practical importance.

The miniaturization of process equipment is one of the fundamental principles of process intensification, which has led to the emergence of microfluidic technology and microchannel reactors [20]. Microchannel reactors utilize characteristic structures at the micrometer or sub-millimeter scale to achieve chemical reactions, resulting in a significant reduction in reactor volume, shortened reaction times, and improved heat/mass transfer efficiency. They represent the forefront and hot topic of chemical engineering research in this century, as well as an important direction for green chemistry development [21]. Microreactors offer several advantages: (1) Precise control of chemical reactions: the micron-sized channels facilitate precise control of reaction temperature and residence time, avoiding differences in reaction processes caused by temperature gradients in conventional batch reactors and improving reaction efficiency [22]. (2) Enhanced safety: the small reaction space within microchannels leads to large physical gradients, enabling rapid heat and mass transfer rates. This effectively removes the heat generated by exothermic reactions and prevents chain reactions, thereby avoiding the common phenomenon of temperature runaway observed in conventional large-scale reactors [23]. (3) Process scaling, flexible debugging, and continuous flow production: system parallel amplification can be achieved through numerical amplification. The reaction conditions and characteristics inside each unit of the microchannel reactor remain unchanged, making it easy to implement modular and flow-based production [24]. Our research team, employing microfluidic technology and 3D printing, has designed and manufactured a series of microchannel reactors. Through finite element simulation, we have studied the mixed flow state of fluids in serrated single channels and subsequently designed a serrated, multi-channel microreactor with a high flow rate. These reactors have been successfully applied in processes such as precious metal and transition metal extraction and separation, as well as nitrogen oxide removal [24,25,26].

Based on the aforementioned research background, this study proposes the utilization of microchannel reactor technology to recover rhodium (Rh) from ROPAC spent catalysts by utilizing the abundant chloride ions present in the catalyst. The recovery process involves the use of H_2_O_2_ as an oxidant in a fully enclosed system. The influence of reaction time, reaction temperature, and H_2_O_2_ dosage on the Rh recovery rate is investigated, followed by response surface methodology (RSM) optimization based on single-factor conditions. Additionally, the reliability of the experiment was verified by Fourier transform infrared (FTIR) and inductively coupled plasma optical emission spectroscopy (ICP-OES) characterization. The objective of this research is to provide a theoretical foundation and industrial innovation for the efficient recovery of Rh from waste catalysts containing Rh.

## 2. Experimental

### 2.1. Materials and Reagents

The ROPAC spent catalyst used in this experiment was obtained from a precious metal recycling company in Yunnan, China. The Rh content in the spent catalyst was determined by microwave digestion using ICP-OES (Agilent. 5110, Santa Clara, CA, USA) with the internal standard method, yielding a concentration of 681.79 mg/L. Additionally, the spent catalyst contained approximately 20% (*v*/*v*) butyraldehyde. Table 1 presents the concentrations of other impurities in ROPAC, including P, Si, Na, Fe, Cr, and Ca. The oxidizing agent used in the experiment was 30% hydrogen peroxide (H_2_O_2_), which was of analytical grade and purity.

### 2.2. Microreactor Structure

**Figure 1:**
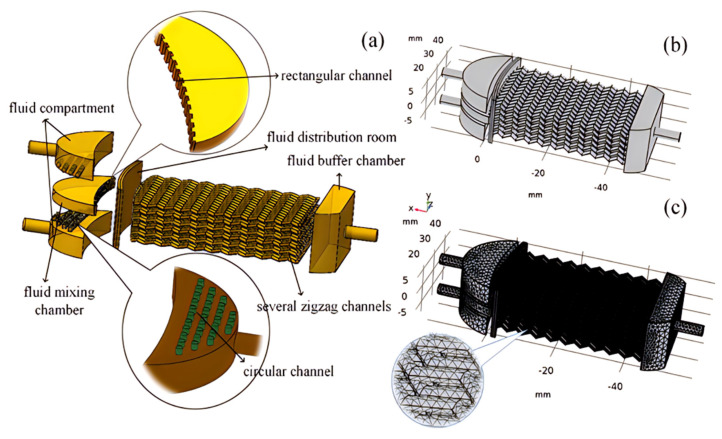
Enlarged schematic diagram of microreactor: (**a**) internal structure diagram; (**b**) 3D printing of the whole large−flow microreactor; (**c**) full view of grid structure [25].

The 3D-printed high-flow microreactors used in this study were all manufactured via 3D printing technology (EOS M290-Mid-Size 3D Printing, Krailling, Germany). The design concept of the 3D-printed high-flow microreactor is based on simulating the mixing flow in a serrated microchannel. The amplification effect is achieved by parallel stacking of multiple serrated microchannels, resulting in a total of 78 microchannels in a single reactor. In addition to the microchannels, the reactor includes fluid mixing chambers, fluid distribution chambers, and interconnecting channels among internal structures, as shown in Figure 1. Oil–water biphasic fluids enter the distribution chamber through the inlet, where wall resistance causes reflux, leading to chaotic flow upon collision with incoming fluids. The chaotic biphasic flow then enters the mixing chamber through inter-wall micropores (Figure 1a). This setup ensures effective mixing of the two phases and prevents stratification due to the effects of chaotic flow. The mixture then passes through serrated microchannels for further mixing (Figure 1b,c). The outer diameter of the reactor’s inlet and outlet is 6 mm, with an inner diameter of 4 mm. The rectangular channels have dimensions of 2 mm (length) and 1 mm (width), while the inner diameter of the serrated channels is 0.6 mm. The microreactor is made of 316 L stainless steel, and stainless steel sleeves are used to connect the inlet and outlet. To ensure sufficient reaction time for the decomposition of H_2_O_2_ and its reaction with the organic phase, a stainless steel extension pipe is designed at the reactor outlet. The extension pipe has an outer diameter of 6 mm, an inner diameter of 4 mm, and a length of 330 mm. The total holding volume of the system is 4.1 L.

### 2.3. Experimental Procedure

The ROPAC spent homogeneous catalyst and H_2_O_2_ were introduced into the microchannel reactor using a co−current pump. The reactor, along with the extension pipe, was placed in a thermostatic oil bath to maintain a constant reaction temperature within the range of 80−120 °C. The flow rate of the co−current pump was controlled to determine the system’s reaction time ratio based on the total fluid volume and the combined flow rate of the two phases. The flow rate of the constant−flow pump was controlled to investigate the organic phase to H_2_O_2_ ratios of 1:0.66−1:2, conducting single-factor experiments. The reaction products were separated using a separating funnel, and the Rh content in the oil phase was analyzed to calculate the Rh recovery rate. The recovery rate of Rh was calculated using the following equations:

(1) $$\mathrm{Recovery}\;\mathrm{efficiency}\;\mathrm{of}\;\mathrm{Rh}=\frac{C_{0}-C_{1}}{C_{0}}\times 100\%$$

where *C*_0_ and *C*_1_ represent the concentrations of Rh before and after the reaction, respectively.

**Figure 2:**
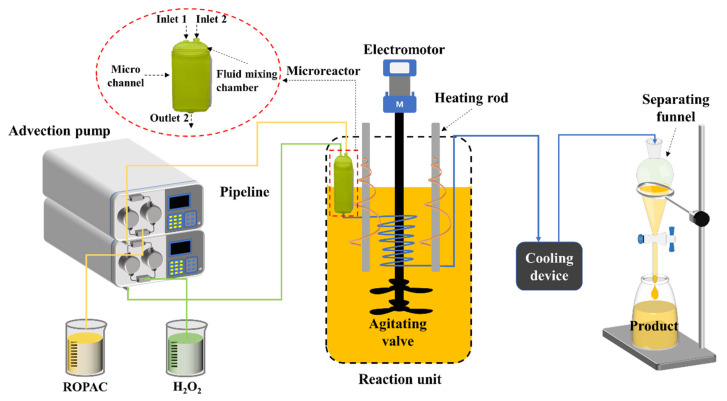
Diagram of the recovery process of spent Rh homogeneous catalysts.

**Figure 3:**
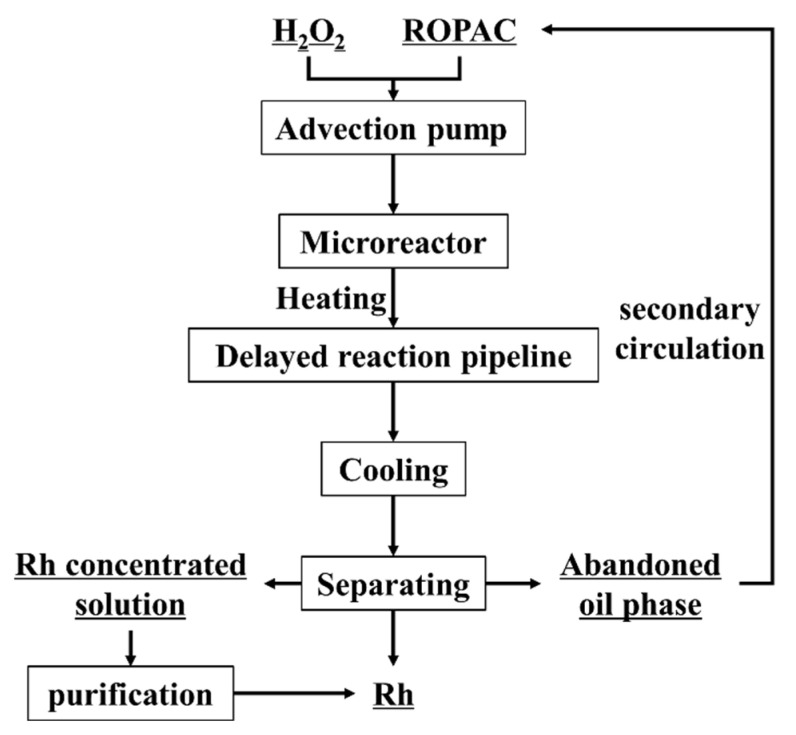
The reaction scheme for the recovery of Rh.

Due to the small internal diameter and long length of the microreactor and extended pipelines, they inherently exhibit a certain degree of capillary resistance. Additionally, ROPAC spent catalyst possesses a certain level of viscosity. Consequently, during the experimental process, the pipeline experiences pressures ranging from 1 to 2 MPa. This high pressure allows the H_2_O_2_ aqueous phase to remain in a liquid state even at temperatures above the normal boiling point, thereby enhancing mass transfer. A schematic of the recovery process of spent Rh homogeneous catalysts is displayed in Figure 2 and Figure 3.

### 2.4. Characterization Methods

The organic compounds in ROPAC spent catalyst and the oil phase of reaction products were analyzed using Fourier transform infrared spectroscopy (FTIR, 640-IR, Varian, Palo Alto, CA, USA). The Rh content in the oil phase of the reaction products was determined by microwave digestion and inductively coupled plasma optical emission spectroscopy (ICP-OES, Agilent. 5110) with an internal standard method.

## 3. Results and Discussion

### 3.1. Effect of Critical Process Parameters on the Recovery Rate of Rh

**Figure 4:**
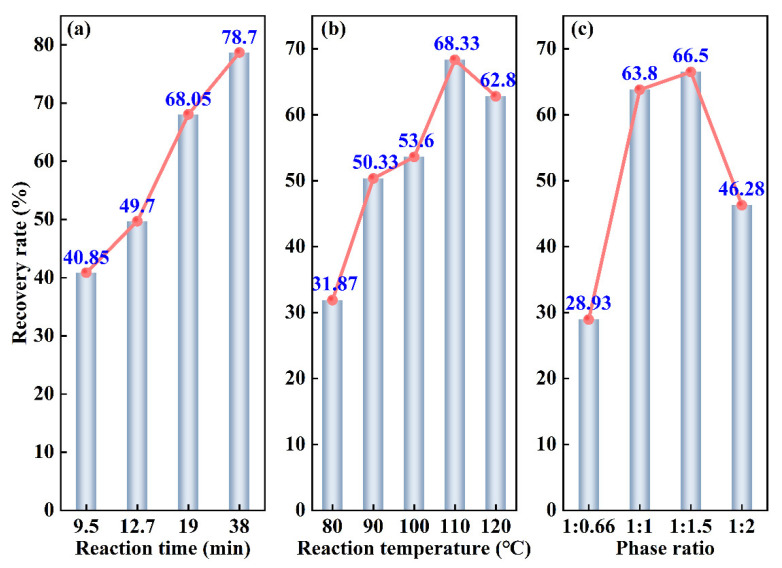
The effect of reaction time (**a**), temperature (**b**), and phase ratio (**c**) on Rh recovery efficiency.

The reaction time, temperature, and phase ratio (volume ratio of ROPAC spent catalyst/H_2_O_2_) are important factors affecting Rh recovery. Therefore, we selected a reaction time of 0–40 min, a temperature of 80–120 °C, and a phase ratio of 1:0.66–1:2 to explore the best experimental conditions. The effect of these three key factors on the Rh recovery rate was investigated, and the specific experimental results are shown in Figure 4.

#### 3.1.1. Reaction Time

Figure 4a shows the trend of Rh recovery rate with respect to reaction time under the conditions of a bath temperature of 110 °C and a volume ratio of 1:1. The reaction time is calculated based on the inlet flow rate of the peristaltic pump and the liquid holding capacity of the system. As the reaction time increases from 9.5 min to 38 min, the Rh recovery rate improves from 40.85% to 78.7%, demonstrating a positive correlation between recovery rate and reaction time. This can be attributed to the rapid release of highly reactive hydroxyl radicals (·OH) from H_2_O_2_ at elevated temperatures. These ·OH radicals combine with the rhodium in the spent catalyst, forming Rh complexes that enter the aqueous phase [27]. Although the microchannel reactor greatly enhances the reaction rate between the oil and water phases, the migration of ·OH at the interface between the two phases is still significantly affected by time. Therefore, a longer reaction time is beneficial for the oxidation process.

#### 3.1.2. Reaction Temperature

The trend of Rh recovery rate with respect to reaction temperature is shown in Figure 4b under the experimental conditions of a fixed reaction time of 19 min and a volume ratio of 1:1. As the temperature increases from 80 °C to 120 °C, the Rh recovery rate initially increases and then decreases. The maximum recovery rate of 68.33% is achieved at a reaction temperature of 110 °C. Increasing the reaction temperature promotes the generation of highly reactive hydroxyl radicals (·OH), leading to more efficient destruction of the triphenylphosphine–rhodium complex and accelerated dissociation of the Rh-P chemical bond [28]. However, excessively high reaction temperatures can cause partial vaporization of H_2_O_2_, resulting in a reduced mass transfer rate at the gas–liquid interface inside the microchannels [29]. Therefore, it is ideal to control the reaction temperature below 110 °C. From a thermodynamic perspective, the recovery of rhodium with H_2_O_2_ is an endothermic and feasible reaction (Rh−C_n_H_m_ + H_2_O_2_ + 3H^+^ + 6Cl^−^→RhCl_6_^3−^ + H−C_n_H_m_ + 2H_2_O). Increasing the temperature will facilitate the forward progress of the reaction. Therefore, raising the reaction temperature appropriately is advantageous for the recovery of rhodium.

#### 3.1.3. Phase Ratio

Figure 4c illustrates the influence of phase ratios on the Rh recovery rate under the optimized conditions of a reaction time of 19 min and a temperature of 110 °C by controlling the constant flow rate of the pump. When the phase ratio is 1:0.66, the Rh recovery rate is only 28.93%, while it increases to 46.28% when the phase ratio is 1:2. This could be attributed to the larger phase ratio, which significantly affects the efficiency of breaking and refining droplets within the microchannel reactor, thereby reducing the mass transfer efficiency at the oil–water interface. With phase ratios of 1:1 and 1:1.5, the Rh recovery rates reach 63.8% and 66.5%, respectively. The initial increase in Rh recovery efficiency with increasing H_2_O_2_ dosage is due to the promotion of highly reactive ·OH generation at low concentrations. This facilitates the destruction of triphenylphosphine rhodium complexes and enhances the dissociation of Rh-P bonds [30]. However, high concentrations of H_2_O_2_ tend to quench ·OH radicals [31], resulting in the formation of ·OH_2_ and a reduction in oxidation efficiency, leading to a plateau in the recovery rate of Rh. Considering cost factors, a phase ratio of 1:1 is more suitable.

In practical industrial production, the spent Rh catalyst is initially introduced into a kettle-type reactor and heated to around 90 °C. Slow addition of H_2_O_2_ with a 1:1 phase ratio is performed with stirring for 8–10 h. Rh enters the aqueous phase in the form of complexes, achieving a recovery rate of up to 95%. The drawback of this process is that the lighter components in the organic phase may slowly evaporate, potentially forming explosive gas when decomposed with hydrogen peroxide, posing a flash explosion hazard. Microreactors, on the other hand, offer the advantage of significantly reducing reaction time while confining the reaction process within sealed microchannels, thus eliminating the risk of flash explosions. To highlight the advantages of the new microreactors process proposed by our studies, the recovery rate comparison between the traditional kettle-type reaction and the microreactor reaction is listed in Table 2.

### 3.2. RSM Optimizes Experimental Design

Based on the results of the single-factor experiments, it is evident that the maximum Rh recovery rate achieved under the optimized process parameters of a reaction time of 19 min, a temperature of 110 °C, and a volume ratio of 1:1 is 68.33%. However, this recovery rate is not satisfactory, and single-factor experiments cannot effectively demonstrate the cross-interactions among factors in the oxidation complex system that influence the Rh recovery rate. Response surface experimental design can simulate the optimization of experimental parameters with a limited number of experiments. By fitting the data, it reveals the mathematical relationship between the Rh recovery rate and the influencing factors, as well as the cross-interactions between them, enabling the optimization of experimental conditions for Rh recovery [32]. Building upon the results of the single-factor experiments, we set ranges for the reaction time (9.5–38 min), reaction temperature (80–120 °C), and oil–water volume ratio (1:0.66–1:2) to establish a response surface model that determines the interaction among these three factors. The Box–Behnken central composite design (BBD) model, with three factors and three levels, is employed to optimize the experimental conditions. Design Expert 13.0 software is used to analyze the interactions between these three factors and their impact on the Rh recovery rate.

#### 3.2.1. Determination of RSM Factors

In RSM, the dependent variable was the recovery rate of Rh, while the independent variables were reaction time, temperature, and phase radio. A mathematical model equation was developed using BBD optimization to predict the optimal process conditions in the recovery process. Table 3 presents the levels and coding of the design factors.

#### 3.2.2. The Recovery Rate Response Surface Experiment and Variance Analysis

Design-Expert software 13 was designed, and the response surface experiment was conducted. The simulation result, real value, and ideal process conditions are all determined by model fitting. The polynomial equation coefficients represent the accuracy and efficacy of the model. The results of the Rh recovery process using the BBD and RSM experimental designs are shown in Table 4.

The following multivariate quadratic response surface regression model is built by Design-Expert software 13 from Table 4:

η(%) = 62.48 + 6.83*A* + 7.65*B* + 4.15*C* − 1.43*AB* − 0.225*AC* − 0.775*BC* − 8.03*A*^2^ − 6.68*B*^2^ − 12.83*C*^2^ (2)

where η represents the response value (Rh recovery rate, %), *A* denotes reaction time, *B* represents reaction temperature, and *C* signifies the phase ratio. The linear factors of *A*, *B,* and *C* contribute positively to the recovery rate, indicating that longer reaction times, higher reaction temperatures, and appropriate phase ratios enhance the recovery rate. Conversely, the interactions among these three factors negatively affect the recovery rate. Thus, when optimizing experimental conditions, it is crucial to consider the combined effects of these factors to achieve the optimal Rh recovery rate. Table 5 shows the experimental findings fitted to the quadratic regression model equation variance analysis.

The model *F*-value is 16.17, and a *p*-value = 0.0007 < 0.0500 implies the model is significant. Values of “*Prob > F*” below 0.0500 indicate significant model terms. In this case, the fitted *R*^2^ = 0.9541, *R*_adj_^2^ = 0.8951, and low C.V.% (7.96 < 15) also suggested that the model fit the experimental data well. *Adeq Precision* measures the signal-to-noise ratio. A ratio greater than 4 is desirable. The *Adeq Precision* of this model is 10.60, indicating that the model is ideal. The *Lack of Fit* value of 0.2507 over 0.05 verified the validity of the model. The analysis of variance reveals that the first-order terms *A*, *B*, and *C* have *Prob > F* values of 0.0018, 0.0009, and 0.0206, respectively, indicating their significant impact on the response variable. On the other hand, the interaction terms *AB*, *AC*, and *BC* show non-significant effects. Moreover, the quadratic terms *A*^2^, *B*^2^, and *C*^2^ are found to have significant impacts. Therefore, in the context of Rh oxidative recovery conditions in this study, the relative influence of each factor on the Rh recovery rate is ranked as follows: temperature > time > volume ratio.

**Figure 5:**
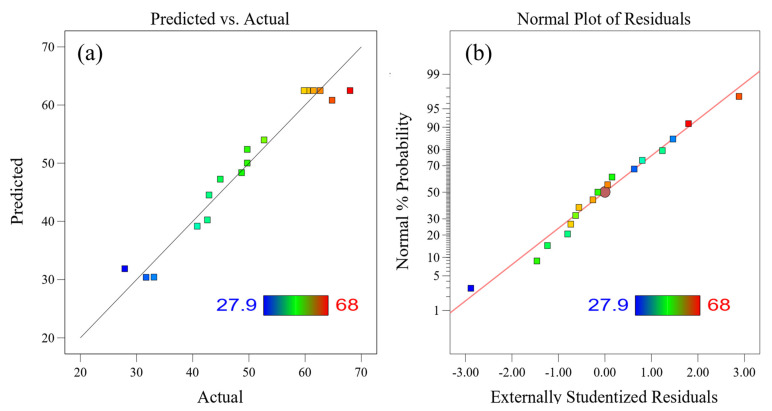
Fitting diagram of the experimental and predicted recovery rate of Rh: (**a**) comparison between the predicted and experimental values; (**b**) experimental residual analysis.

Figure 5a presents a comparison between the predicted and experimental findings of the quadratic regression model for the recovery of Rh. The plot showed a linear representation of predicted model values with scattered dots deviating from the line, confirming the validity of the quadratic regression model. The closer the points are to the line, the better the fitting effect. Figure 5b displays the results of the experimental residual analysis, indicating a virtually straight and outlier-free distribution of the experimental residual values. As a result, the selected model demonstrates soundness, close alignment of experimental results with expected values, and effective capture of the relationship between factors and response values [33].

#### 3.2.3. Response Surface Analysis, Optimization, and Verification

**Figure 6:**
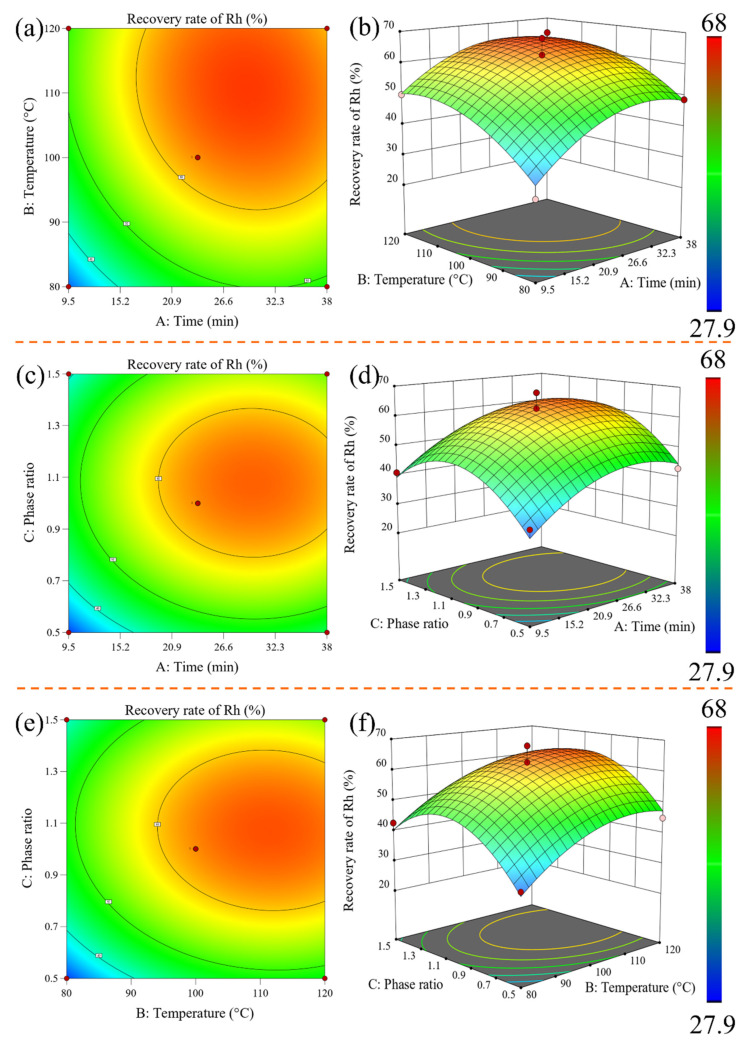
Contour map and response surface graph of the interaction among three factors on the recovery rate of Rh: contour map (**a**) and response surface map (**b**) show how temperature, time, and their interactions influence the recovery rate; contour diagram (**c**) and response surface diagram (**d**) display how phase ratio, time, and their interactions influence the recovery rate; contour diagram (**e**) and response surface diagram (**f**) exhibit phase ratio, temperature, and their interaction on the recovery rate.

The contour plot and response surface plot depicting the interaction effects of the factors on the Rh recovery rate are presented in Figure 6, generated using Design-Expert 13 software. In the response surface plot, the projection of the contours falling in the horizontal direction represents the contour lines. If the contour lines form elliptical shapes, it indicates a highly significant interaction between the two factors. Conversely, if the contour lines form circular shapes, it suggests that the interaction is not significant [34]. Additionally, the steepness of the slopes on the response surface plot reflects the degree of influence of each factor on the Rh recovery rate. A steeper slope indicates a greater impact [35]. Based on the analysis of the contour plot and response surface plot, it can be concluded that the interaction between time and temperature has the most significant influence on the Rh recovery rate. The red region on the surface plot represents Rh recovery rates greater than 60%, the yellow region represents recovery rates between 50% and 60%, and the green region indicates recovery rates below 50%. When *A* and *B* are fixed at 23.75 min and 100 °C, respectively, as *A* increases from 0.5 to 1.5, the Rh recovery rate initially increases and then decreases. Similarly, when *A* and *C* are fixed at 23.75 min and 1, respectively, as *B* increases from 80 °C to 120 °C, the Rh recovery rate continues to increase. Furthermore, when *B* and *C* are fixed at 100 °C and 1, respectively, the Rh recovery rate shows an initial sharp increase followed by a slight decrease as the *A* value increases. Through the simulation analysis of Design-Expert 13.0 software, the optimal parameters for the maximum Rh recovery rate can be obtained: the reaction time is 29 min, the reaction temperature is 110 °C, and the phase ratio is 1. In order to reduce the experimental error, three sets of parallel experiments were designed to verify the optimal conditions for the prediction of the quadratic regression response model. The results are shown in Table 6. It can be seen from Table 6 that the recovery rate of Rh was between 65.85 and 66.10% under the optimal process conditions predicted by the model, and the average value was 65.98%. The theoretical prediction value is 66.06%. It can be seen that the experimental value is very close to the predicted value, and the relative deviation is only 0.08% (<5%), indicating that the optimal process conditions obtained by using the quadratic regression response model to predict and optimize are reliable. In order to further improve the Rh recovery rate, a continuous experiment was conducted for 4 h using the optimized parameters, resulting in an Rh recovery rate of 74.93%. This exceeds the predicted Rh recovery rate of 66.06% and thus achieves the goal of process optimization.

Compared to the traditional batch reactor process, which typically requires 8–10 h of processing time [36], the microreactor-based treatment process significantly reduces the processing time while enabling continuous production. However, the greatly reduced reaction time may limit the extent of the reaction. Therefore, a two-stage treatment approach is considered to compensate for the shortened reaction time. This involves subjecting the organic phase recovered from the first stage to a secondary treatment under the same process conditions. The results from a continuous two-stage production experiment conducted for 4 h show an overall Rh recovery rate of 95.6%, achieving a highly desirable recovery effect.

### 3.3. FTIR Evidence of the Rh Recovery Mechanism

**Figure 7:**
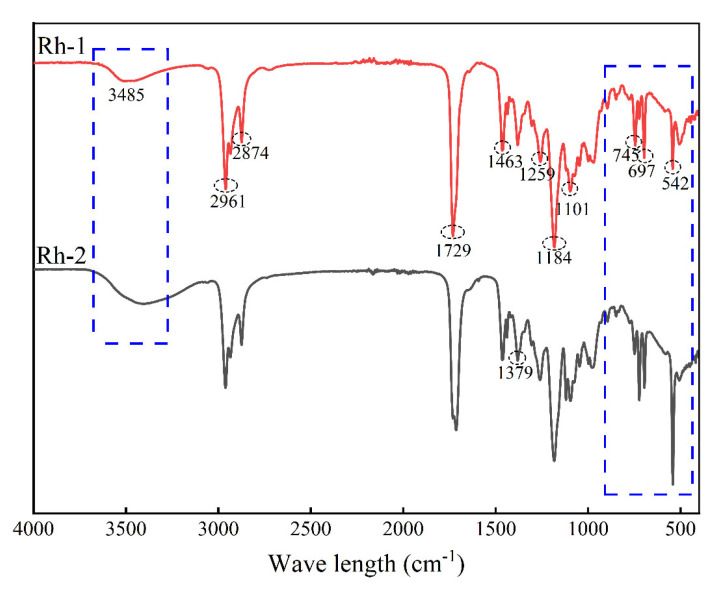
Infrared spectrum of waste homogeneous catalyst before and after treatment. (Rh−1) Waste homogeneous catalyst; (Rh−2) Product oil phase.

To determine the mechanism of the separation system, the structural changes in the organic reagents that occurred during the reactions were analyzed with FTIR spectroscopy. The infrared spectroscopy analysis results of the ROPAC spent homogeneous catalysts are shown in Figure 7, where Rh-1 and Rh-2 represent the infrared spectra of the ROPAC spent catalyst before and after oxidation treatment, respectively. By comparative analysis, it can be observed that the absorption peak at 3485 cm^−1^ corresponds to the O-H stretching vibration [37]. The significant increase and broadening of this absorption peak indicate the presence of -OH entering the oil phase during the reaction. The strong absorption peaks at 2961 cm^−1^, 2874 cm^−1^, 1463 cm^−1^, and 1379 cm^−1^ correspond to alkane groups and aliphatic moieties (such as CH_2_, C-H, C=C, and C-C) [38]. The absorption peaks at 1184 cm^−1^, 1101 cm^−1^, and 697 cm^−1^ mainly arise from the stretching vibrations of P=O and C-O functional groups [39,40], suggesting the presence of some free alcohols in the heterogeneous catalyst, with minimal changes before and after the reaction. The characteristic bands at 1729 cm^−1^, 1259 cm^−1^, and 745 cm^−1^ are attributed to carbonyl C=O stretching, aromatic rings, and P-C stretching vibrations, respectively [41,42]. The absorption peak at 542 cm^−1^ corresponds to the stretching vibration of C-Cl, which exhibits significant enhancement after the reaction, indicating the presence of a certain amount of chloride ions in the spent catalyst, with some remaining in the oil phase. Considering the operating environment of the ROPAC catalyst, it is speculated that the spent catalyst consists of alcohols, aldehydes, saturated alkanes, triphenylphosphine, and triphenylphosphine oxide.

**Figure 8:**
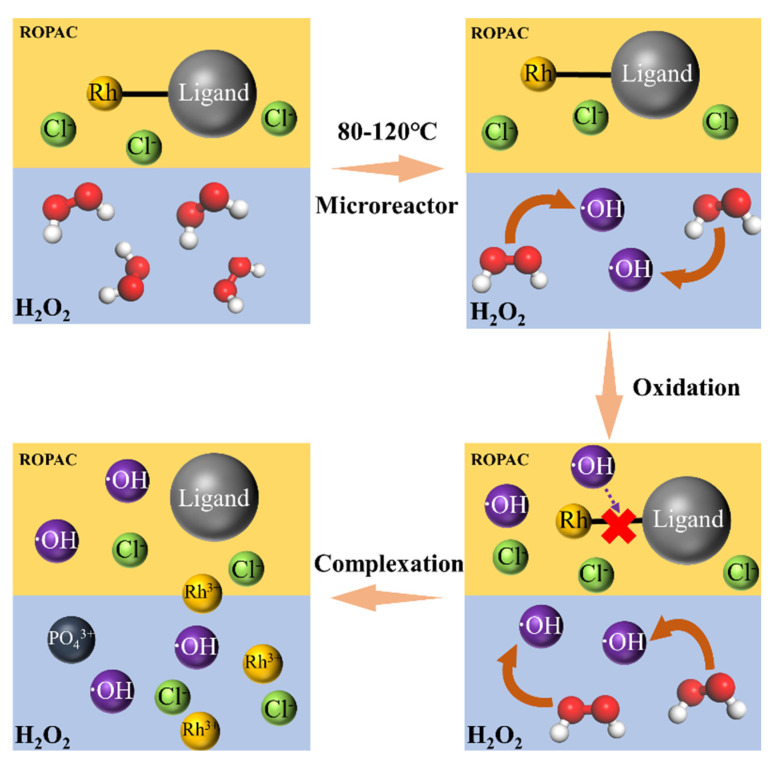
Diagram of oxidation recovery of Rh using spent homogeneous catalysts.

The analysis using ICP-OES on the reaction products reveals that the concentration of Rh in the organic phase is 29.99 mg/L, while the concentration of chloride (Cl^−^) in the aqueous phase is 209.33 mg/L, and the phosphate (PO_4_^3+^) concentration is 266.01 mg/L. These results indicate that the recovery of Rh from the spent catalyst has been achieved almost completely. Additionally, the presence of a relatively high concentration of chloride in the spent catalyst suggests that it accompanies Rh into the aqueous phase, forming rhodium–chloride water complexes. The appearance of PO_4_^3+^ may be attributed to the partial oxidation and degradation of triphenylphosphine structures during the operation of the ROPAC catalyst or during the oxidative recovery process. Therefore, the process of Rh oxidation recovery can be divided into the following four steps, as shown in Figure 8. Additionally, it is important to note that PO_4_^3+^ exhibits strong corrosive properties towards metallic materials at elevated temperatures. This corrosion potential can lead to equipment corrosion or even unexpected side reactions. Further research will be carried out to validate these effects and evaluate their potential impact on the overall process.

## 4. Conclusions

This paper presents a study on the waste homogeneous catalyst ROPAC, utilizing microchannel reactors as the experiment setup. The effects of reaction time, temperature, and phase ratio on the recovery of Rh were investigated. Furthermore, RSM was utilized for the optimization of process parameters, encompassing reaction time, temperature, and phase ratio. Lastly, the reaction mechanism involved in the oxidation and recovery of Rh was elucidated using FTIR and ICP-OES analysis. The primary findings can be summarized as follows:

(1) Using microchannel reactor technology as a replacement for traditional processes in the oxidative recovery of waste rhodium homogeneous catalysts has shown significant effectiveness. This approach demonstrates remarkable improvements in reaction efficiency and process time reduction while also offering notable advantages over traditional methods in terms of environmental friendliness, sustainability, and safety considerations.
(2) By oxidatively breaking the Rh-P chemical bond using H_2_O_2_, the complexation recovery of Rh was achieved. Based on the single-factor experiment, response surface optimization design was applied to obtain the optimized process conditions: reaction time of 29 min, reaction temperature of 110 °C, and phase ratio of 1:1. The results of the continuous experiment for 4 h showed a Rh recovery rate of 74.93%. Under the same conditions, secondary oxidation treatment resulted in a Rh recovery rate of 95.6%.
(3) Under the optimized conditions, the Rh content in the oil phase was measured at 21.22 mg/L, while the concentration of chloride (Cl^−^) in the aqueous phase is 209.33 mg/L and the phosphate (PO_4_^3+^) concentration is 266.01 mg/L. These findings indicate the near-complete recovery of Rh from the spent catalyst. The removal mechanism of Rh in ROPAC can be further confirmed through FTIR analysis. Simultaneously, the higher concentration of chlorine in the waste catalyst indicates the entry of rhodium into the aqueous phase, forming a rhodium chloride aqueous complex.

## Figures and Tables

**Table 1:** The main chemical composition of ROPAC spent homogeneous catalyst (mg/L).

Elements	Ca	Cr	Fe	Na	P	Rh	Si
Content	3.47	3.50	9.68	14.92	4320.99	681.79	21.27

**Table 2:** Comparison of different treatment methods for the recovery rate of Rh.

Methods	Temperature (°C)	Time (h)	Phase Ratio	Recovery Rate (%)	Security
Kettle-type reactor	90	>8.00	1:1	≤95%	The danger of deflagration gas overflow
Microreactors	110	0.66	1:1	95.6%	Safe

**Table 3:** Level and coding of factors.

Factors	Codes	Levels		
		−1	0	+1
Time/min	A	9.5	23.75	38
Temperature/°C	B	80	100	120
Phase radio	C	0.5	1	1.5

**Table 4:** Design of the response surface experimental scheme.

	Factor 1	Factor 2	Factor 3	Response Value
Run	A: Time/min	B: Temperature/°C	C: Phase Radio	η/%
1	23.75	100.00	1.00	69.8
2	9.5	120.00	1.00	59.7
3	38.00	80.00	1.00	58.7
4	23.75	80.00	1.50	52.6
5	23.75	100.00	1.00	72.7
6	9.50	80.00	1.00	37.9
7	23.75	100.00	1.00	78
8	23.75	120.00	0.50	54.9
9	23.75	100.00	1.00	71.5
10	23.75	100.00	1.00	70.4
11	38.00	100.00	1.50	59.7
12	23.75	80.00	0.50	41.7
13	38.00	120.00	1.00	74.8
14	38.00	100.00	0.50	52.9
15	9.50	100.00	1.50	50.8
16	9.50	100.00	0.50	43.1
17	23.75	120.00	1.50	62.7

**Table 5:** Analysis of variance table for response surface quadratic model.

Source	SS	DF	MS	*F*-Value	*p*-Value (Prob > *F*)	Significance
Model	2261.99	9	251.33	16.17	0.0007	significant
*A*	372.65	1	372.65	23.97	0.0018	
*B*	468.18	1	468.18	30.12	0.0009	
*C*	137.78	1	137.78	8.86	0.0206	
*AB*	8.12	1	8.12	0.5225	0.4932	
*AC*	0.2025	1	0.2025	0.0130	0.9123	
*BC*	2.40	1	2.40	0.1545	0.7059	
*A* ^2^	271.33	1	271.33	17.45	0.0041	
*B* ^2^	187.74	1	187.74	12.08	0.0103	
*C* ^2^	692.82	1	692.82	44.57	0.0003	
Residual	108.82	7	15.55			
Lack of Fit	65.83	3	21.94	2.04	0.2507	not significant
Pure error	42.99	4	10.75			
Cor total	2370.81	16				

SS—Sum of squares; DF—Degrees of freedom; MS—Mean square. *R*^2^ = 0.9541, *R*_adj_^2^ = 0.8951, C.V.% = 7.96, *Adeq Precision* = 10.6086.

**Table 6:** Verification of predicted value of quadratic regression response model.

Time/min	Temperature/°C	Phase Radio	Rh Recovery Rate/%
29	110	1	66.00
29	110	1	65.85
29	110	1	66.10

## Data Availability

Not applicable.
